# RNA interference approaches for treatment of HIV-1 infection

**DOI:** 10.1186/s13073-015-0174-y

**Published:** 2015-05-28

**Authors:** Maggie L Bobbin, John C Burnett, John J Rossi

**Affiliations:** Irell & Manella School of Biological Sciences, Beckman Research Institute of City of Hope, East Duarte Road, Duarte, CA 91010 USA; Department of Molecular and Cell Biology, Beckman Research Institute of City of Hope, East Duarte Road, Duarte, CA 9101 USA

## Abstract

HIV/AIDS is a chronic and debilitating disease that cannot be cured with current antiretroviral drugs. While combinatorial antiretroviral therapy (cART) can potently suppress HIV-1 replication and delay the onset of AIDS, viral mutagenesis often leads to viral escape from multiple drugs. In addition to the pharmacological agents that comprise cART drug cocktails, new biological therapeutics are reaching the clinic. These include gene-based therapies that utilize RNA interference (RNAi) to silence the expression of viral or host mRNA targets that are required for HIV-1 infection and/or replication. RNAi allows sequence-specific design to compensate for viral mutants and natural variants, thereby drastically expanding the number of therapeutic targets beyond the capabilities of cART. Recent advances in clinical and preclinical studies have demonstrated the promise of RNAi therapeutics, reinforcing the concept that RNAi-based agents might offer a safe, effective, and more durable approach for the treatment of HIV/AIDS. Nevertheless, there are challenges that must be overcome in order for RNAi therapeutics to reach their clinical potential. These include the refinement of strategies for delivery and to reduce the risk of mutational escape. In this review, we provide an overview of RNAi-based therapies for HIV-1, examine a variety of combinatorial RNAi strategies, and discuss approaches for *ex vivo* delivery and *in vivo* delivery.

## Introduction

**Fig. 1:**
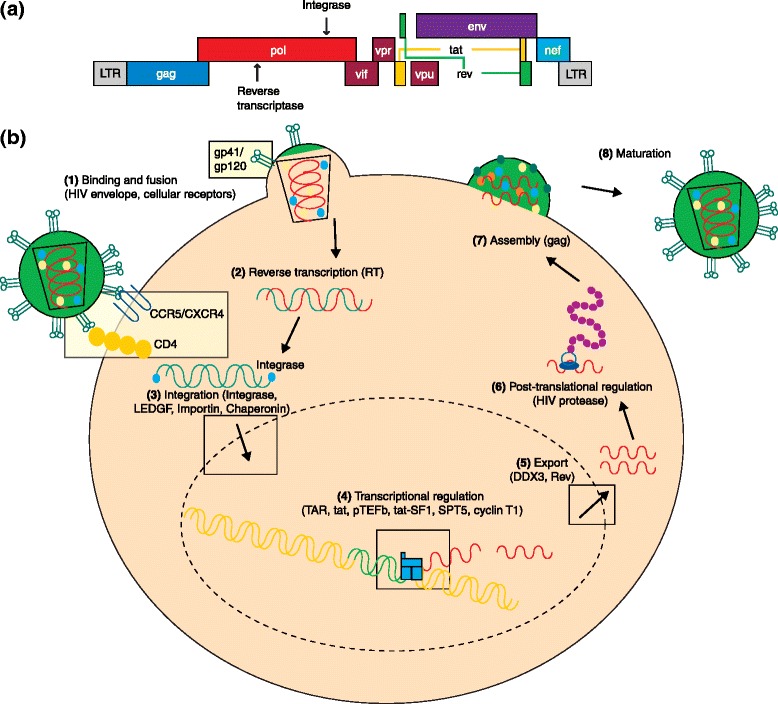
The HIV-1 genome and strategies for antiviral targeting. **a** Structure of the HIV-1 genome. The genome contains nine genes and two long terminal repeats (LTRs) that can be targeted by RNA interference (RNAi). Certain genomic regions are more conserved than others, making them better targets. In addition, many of the genes are alternatively spliced, requiring careful target design. **b** HIV-1 targeting. Several steps of the HIV-1 viral replication cycle can be targeted by RNAi. Current drug targets are in parentheses. (1) The first step is receptor binding and membrane fusion by the HIV envelope glycoproteins gp120 and gp41 to host receptors CD4 and either CCR5 or CXCR4. This step can be inhibited by knocking down the HIV-1 co-receptors, CCR5 or CXCR4. (2) Next, the viral genome must be reverse transcribed by the viral reverse transcriptase (RT) and (3) integrated into the cellular genome which is mediated by the viral integrase protein and host factors LEDGF, Importin, and Chaperonin. After integration, (4) the virus is transcribed, which is mediated by viral (TAR and tat) and host (pTEFb, tat-SF1, SPT5, cyclin T1) factors, (5) exported to the cytoplasm (dependent on DDX3 and Rev) and then translated and (6) subjected to post-translational processing by the viral protease. (7) Finally, the proteins are processed and (8) packaged into new viral particles

The genome of the human immunodeficiency virus-1 (HIV-1) is composed of nine viral genes (*gag*, *pol*, *vif*, *vpr*, *tat*, *rev*, *vpu*, *env*, and *nef*) that are required for all processes in the viral replicative cycle, including viral assembly, viral entry and receptor binding, membrane fusion, reverse transcription, integration, and proteolytic protein processing (Fig. 1). HIV-1 preferentially infects human CD4+ T-lymphocytes, eventually leading to a depletion of CD4+ cells and the clinical progression to acquired immunodeficiency syndrome (AIDS). Since the discovery of the antiretroviral activity of azidothymidine (AZT) in 1985 [1], a variety of antiretroviral drugs have been developed that target multiple steps in the viral replication cycle (Table 1). Typically, a cocktail of three antiretroviral drugs is used as a combinatorial antiretroviral therapy (cART) that can effectively suppress viral replication, reduce rates of transmission, and improve patients’ life expectancy by prolonging the onset of AIDS [2]. Nevertheless, cART does not fully restore health, as patients on cART still experience chronic inflammation and increased rates of non-AIDS morbidity and mortality [3]. Moreover, current cART regimens are unable to cure HIV-1 patients, as these drugs cannot eradicate latent viral reservoirs and may also fall short in completely suppressing viral replication despite drug intensification [4–7]. Thus, nearly all HIV-infected individuals on cART will need to maintain their medications for the entirety of their lives, resulting in considerable expense, development of resistance, and toxic side effects. Owing to improvements in the efficacy and availability of antiretroviral drugs and efforts in HIV/AIDS prevention, recent years have seen a decrease in the incidence of HIV-1 infection. Nevertheless, there are still 35.3 million people living with HIV-1, with 2.3 million new cases and 1.6 million deaths in 2012 [8]. Because of the limitations and adverse effects associated with current cART regimens, it is necessary to develop alternative therapeutic strategies that are safer, more efficacious, and more resistant to viral escape. Such emerging therapeutic strategies include gene-based and nucleic-acid-based therapies that are based on gene editing, ribozymes, and RNA interference (RNAi).

**Table 1:** Current therapies, clinical trials, and strategies to combat HIV

Therapeutic approach	Company/institute	Component(s)	Target(s)	Delivery	Progress	NCT identifier	Reference(s)
Combinatorial anti**-**retroviral therapy (cART)	Gilead, Merck, GSK, Pfizer, Tibotec, Abbott, Bristol-Meyers Squibb, Hoffman-La Roche	1. CCR5 antagonist	1. Receptor binding	*In vivo* drug administration	Current method to treat HIV		[159]
		2. Fusion inhibitors	2. Membrane fusion				
		3. Nucleoside analogs or RT inhibitors	3. Reverse transcription				
		4. Strand transfer inhibitor	4. Integration				
		5. Protease inhibitors	5. Protein processing				
Peptide or protein vaccines	VaxGen, Merck, NIH/NIAID	Gp120	Viral envelope	*In vivo* injection	Phase I/II/III	NCT00001031	[160–162]
		Gp41/160				NCT00002402	
		Gag/pol/nef	Viral proteins			NCT00223080	
						NCT01435135	
						NCT00002441	
Inhibitory peptide	University Medical Center Hamburg-Eppendorf	C46 anti-viral peptide	Viral fusion to cell membrane	*Ex vivo* retroviral modified CD4+ cells	Phase I		[163]
Drug shRNA peptide	Calimmune/Caltech/UCLA	1. Bisulfide	1. Transplant conditioning	*Ex vivo* lentiviral modified CD34+ and CD4+ cells	Phase I/II	NCT01734850	[139]
		2. CCR5 shRNA	2. Host co-receptor				
		3. C46 peptide	3. Viral envelope				
shRNA ribozyme RNA decoy	Benitec/City of Hope	1. tat/rev shRNA	1. Viral mRNA	*Ex vivo* lentiviral modified CD34+ cells	Phase I	NCT00569985	[38, 39]
		2. CCR5 ribozyme	2. Host co-receptor				
		3. TAR decoy	3. Viral Tat protein				
Zinc finger nuclease	Sangamo Biosciences	1. CCR5 zinc finger nuclease	CCR5 DNA	*Ex vivo* AAV modified autologous CD4+ T cells	Phase I-II	NCT00842634	[20]
						NCT01044654	
						NCT01543152	
						NCT02225665	
Ribozyme	Janssen-Cilag Pty Ltd, UCLA	tat-vpr anti-HIV ribozyme	Tat-vpr mRNA	*Ex vivo* retroviral modified autologous CD34+ HPC	Phase I/II	NCT00074997	[40, 41]
						NCT01177059	
Ribozyme	UCSD	CCR5 ribozyme	CCR5 mRNA	*Ex vivo* retroviral modified autologous CD4+ T cells	Phase I	FDA BB-IND 6405	[42]
Ribozyme	Johnson & Johnson, St Vincent’s Hospital	Tat anti-HIV ribozyme	Translation initiation mRNA of Tat	*Ex vivo* retroviral modified syngeneic CD4+ T cells	Phase I	NCT00074997	[43]
Ribozyme	Ribozyme, City of Hope	Tat anti-HIV ribozyme	Tat-rev mRNA	*Ex vivo* retroviral modified CD34+ HPC	Phase II	NCT00002221	[164]
Antisense	VIRxSYS Corporation	937 base antisense gene	Env mRNA	*Ex vivo* lentiviral (LTR HIV) modified CD4+ cells	Phase I/II	NCT00131560	[165]
Antisense	Enzo Biochem	U1/HIV anti-sense RNA (61–68 bases)	TAR, tat/rev	*Ex vivo* retroviral modified CD34+ HPC	Phase I/II	NCT00003942	[166]
						NCT00001535	
RNA decoy	Children’s Hospital Los Angeles	Rev response element decoy	Rev protein	*Ex vivo r*etroviral modified CD34+ HPC	Phase 0- pilot		[167]

*Abbreviations*: AAV, adeno-associated virus; HPC, human progenitor cells; LTR, long terminal repeat; NCT, National Clinical Trial; NIH/NIAID, National Institutes of Health/National Institute of Allergy and Infectious Diseases; RT, reverse transcriptase; shRNA, short hairpin RNA

Like cART, gene-therapy strategies against HIV-1 are often designed to target multiple viral mechanisms, which reduces the possibility of viral-escape-induced drug resistance and improves therapeutic efficacy. This is particularly true for RNAi therapeutics, as small interfering RNAs (siRNAs) and short hairpin RNAs (shRNAs) can be designed to target conserved 21-nucleotide sequences within the 9.2-kb HIV-1 genomic RNA, thereby expanding the possible targets far beyond those of current drugs. Moreover, multiplexing siRNAs or shRNAs as a single therapy can increase the overall potency and reduce the dosage of each component required to minimize off-target toxicity.

This review highlights recent advances in gene-therapy technologies for the treatment and eradication of HIV-1. Many new therapies are currently in development with the hope of preventing drug resistance, toxicity, and latent infection. Owing to their design flexibility and target specificity, we pay particular attention to RNAi-based therapies that target viral or host targets, including some that have reached human clinical testing. Delivery of the RNAi agents to HIV-infected or HIV-susceptible cells remains a challenging problem that is currently being addressed by *ex vivo* delivery, targeted *in vivo* delivery, and non-targeted *in vivo* delivery strategies.

## HIV-1 infection and therapeutic targets

HIV-1 gains entry into CD4-expressing T-lymphocytes, macrophages, and monocytes by fusion of the viral envelope with the host cell membrane (reviewed in [9]). The HIV-1 envelope is characterized by trimeric spikes, and each spike contains three glycoprotein 120 (gp120) surface subunits and three gp41 trans-membrane subunits. The process of viral binding and entry into the cell generally requires the CD4 receptor and either the CCR5 or the CXCR4 co-receptor. Upon receptor-mediated binding and internalization, the viral RNA genome is converted to double-stranded DNA, using reverse transcriptase that is provided by the virus. The viral genomic DNA is then imported into the nucleus and integrated into the host genome, where it is transcribed into the full-length 9.2-kb mRNA transcript. This transcript can undergo double splicing into 1.8-kb mRNAs encoding Tat, Rev, and Nef proteins. Tat and Rev travel to the nucleus where Tat activates transcription and Rev mediates the export of mRNAs in the late phase. Singly-spliced 4.0-kb transcripts encoding Env, Vpu, Vpr, and Vif proteins [10] and unspliced 9.2-kb mRNA transcripts encoding Gag and Gag-Pol are exported from the nucleus in a Rev-dependent manner. These unspliced mRNAs also function as the genomic RNA for progeny virus. Several Gag molecules bind to the viral genome and are trafficked to lipid rafts on the plasma membrane of the host cell [11, 12]. The viral genome is assembled as a dimer with viral proteins and exits the cell via budding. Final maturation occurs once the viral particles are released from the host cell. These steps provide many opportunities for therapeutic intervention.

Owing to the limitations of current cART approaches, alternative gene- and nucleic-acid-based therapies have been proposed to control HIV-1 infection, including gene editing, ribozymes, and utilization of RNA decoys. In addition, gene- and nucleic-acid-based agents have been developed as HIV-1 prophylactics, including RNA aptamers and siRNAs that are used as microbicides and viral genes used as vaccines (Table 1).

Gene editing, which requires a sequence-specific nuclease that modifies a target gene, is a particularly attractive approach that aims to develop cells that are permanently resistant to HIV-1 infection or to eliminate the HIV-1 provirus from infected cells [13]. HIV-resistant cells can be established by genomic editing of the *CCR5* or *CXCR4* co-receptors using zinc finger nucleases (ZFNs) [14–21], transcription activator-like effector nucleases (TALENs) [22], designer endonucleases [23], peptide nucleic acids (PNAs) [24], or CRISPR/Cas9 [22, 25, 26]. Alternatively, the integrated HIV-1 genome in infected cells can be targeted and destroyed using CRISPR/Cas9 [27, 28], homing endonucleases [29], and HIV-1 long terminal repeat (LTR)-specific recombinases [30–32]. Catalytic RNAs, known as ribozymes, can complete a similar task, but the effects are not permanent because their target molecule is mRNA [33]. RNA aptamers and decoys can bind viral proteins to act as steric or competitive inhibitors [34–36]. By combining an anti-HIV-1 microRNA (miRNA) with a TAR decoy (TARmiR), a dual-functioning RNA molecule can suppress HIV-1 expression by RNAi and also bind to the HIV-1 transactivating protein Tat, reducing the numbers of Tat molecules that can bind to its cognate target TAR to transactivate viral transcription [37]. Several of these technologies have reached clinical testing as single or combinatorial HIV-1 therapies [2], including CCR5 ZFN [20], CCR5 ribozymes [38, 39], HIV-1 ribozymes [40–43], and TAR decoys [38, 39].

## Endogenous RNA silencing pathway

**Fig. 2:**
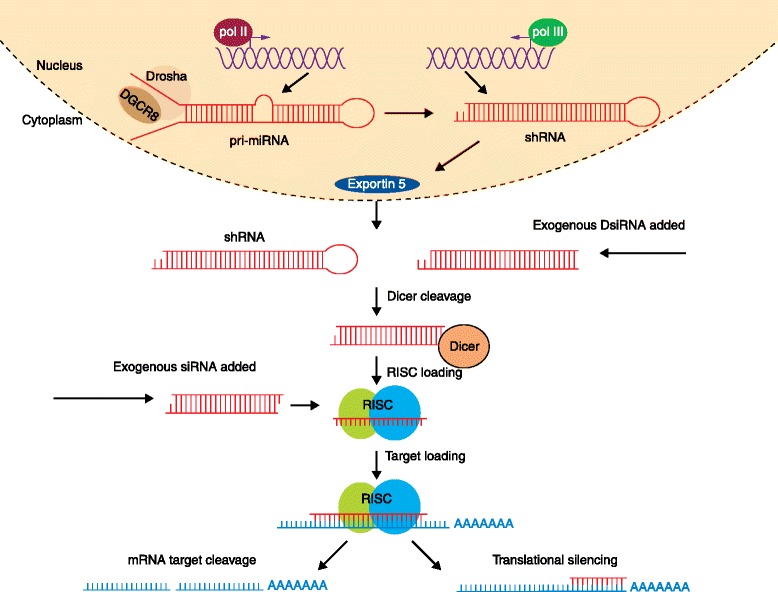
RNA silencing mechanism. For therapeutic RNAi applications, a lentiviral vector or plasmid is transcribed by RNA pol III into shRNA. This shRNA is then exported from the nucleus to the cytoplasm by Exportin 5. After export, shRNA or miRNA are processed by Dicer and loaded into RISC. For exogenously delivered siRNA or Dicer substrate RNA (dsiRNA), the processed siRNA is loaded directly into RISC. The passenger strand is removed from the guide strand and RISC guides the remaining strand to its complementary mRNA target. RISC then cleaves the mRNA for degradation. Abbreviations: pol III, RNA polymerase III; pol II, RNA polymerase II; RISC, RNA-induced silencing complex; shRNA, short hairpin RNA; siRNA, short interfering RNA

RNAi is a natural cellular mechanism first identified by Craig Mello and Andrew Fire in 1998, which involves the pairing of a short miRNA sequence to an endogenous mRNA target [44]. Endogenous miRNAs regulate many cellular mechanisms and are processed in a very specific manner. First, double-stranded miRNAs are loaded into the RNA-induced silencing complex (RISC), where the non-targeting (passenger) strand is removed. The RISC complex then finds the complementary mRNA sequence for the guide strand of the miRNA. The binding of the miRNA to the mRNA target results in gene silencing through degradation of the mRNA (Fig. 2). While the canonical RNAi pathway occurs in the cytoplasm, RNAi can also function in the nucleus to induce gene-specific transcriptional gene silencing or transcriptional gene activation by directing epigenetic changes in gene promoters [45–47]. Teasing out these mechanisms has resulted in a better understanding of how mammalian cells use this system to regulate endogenous gene expression.

While the role of the RNAi system in mammalian cells is well documented (reviewed in [48]), the role of mammalian RNAi in response to viral infections is still the subject of much debate [49]. It is well established that viral RNAs trigger innate interferon (IFN) responses in mammalian cells as a first line of defense. While viral small RNAs (vsRNAs) have been detected in infected mammalian cells [50], it was previously uncertain whether vsRNAs could function via an antiviral RNAi pathway. One reason for this uncertainty is that many viruses encode viral suppressors of RNAi (VSRs), which are viral proteins that inhibit the host RNAi pathway [51, 52]. Two important studies from 2013, which examined conditions that are unfavorable for VSRs, reported that vsRNAs act via an RNAi pathway [53, 54]. Encephalomyocarditis virus infection of undifferentiated mouse embryonic stem cells, which have low or no innate IFN response, resulted in the accumulation of virus-derived siRNAs (vsiRNAs) that associated with Argonaute-2 (Ago2) [53]. vsiRNAs were not, however, observed in differentiated cells, which have high innate IFN activity. Thus, antiviral RNAi appears to be most prevalent in conditions that are unfavorable for innate IFN viral clearance. Similarly, infection by a Nodamura virus strain that contained a mutant VSR protein was attenuated because host cells produced vsRNAs in response to infection with these mutant viruses [54]. Despite these compelling reports, seemingly contradictory observations have recently been reported that indicate that viral RNAi does not occur in Dicer-knockout cells or in cells with disabled IFN function [55, 56]. Thus, the potential role of antiviral RNAi in mammalians remains controversial and appears to be highly dependent on the host cells and the particular virus.

The RNA silencing pathway provides a tool for gene therapy by the delivery of artificial RNAi in the form of a small RNA duplex, such as a miRNA, siRNA, Dicer substrate RNA (DsiRNA), or shRNA [57]. Although siRNA, DsiRNA, and shRNA all function through the same RNAi pathway, shRNA requires additional processing in the nucleus (Fig. 2). shRNAs differ from siRNAs because they comprise a single strand of RNA with two complementary tails connected by a hairpin loop, and are commonly transcribed from viral vectors or plasmid DNA in the nucleus. Following transcription, the shRNA is exported to the cytoplasm, where it is processed in the same manner as siRNA by Dicer in complex with TRBP, a member of the RISC. Alternatively, Dicer-independent Ago2-shRNAs are shRNAs that have a short stem length and are incorporated into RISC without Dicer processing [58]. After incorporation into RISC, the processed siRNA is loaded onto an mRNA target to induce either mRNA cleavage by Ago2 or translational repression and subsequent mRNA degradation.

Toxicity of RNAi can occur through improper target recognition resulting from suboptimal annealing of the antisense guide strand to the target strand, improper selection of an unintended passenger strand by the RISC complex, or overloading of the silencing machinery. Biasing the strand-selection of the guide strand, rather than of the passenger strand, can enhance potency while also reducing off-target effects of the passenger strand. Strand selection can be biased by designing asymmetric siRNAs with less stable thermodynamic stability at the 5′ end of the guide strand [59, 60], by chemical modifications at one or both ends of the siRNA [61, 62], or by the design of structurally asymmetric shRNAs or siRNAs that have a single 2-nucleotide 3′-overhang on the guide strand [63–66]. One of the most reliable design strategies for generating strand-biased siRNAs is to use asymmetric 25/27-nucleotide DsiRNAs [64, 65]. After processing by Dicer, the guide strand from the asymmetric 25/27mer DsiRNA is preferentially incorporated into the RISC, although the siRNA potency can still be influenced by thermodynamic stabilities at both ends [67, 68].

In addition, overloading can be avoided by limiting the amounts of exogenous RNA added or by using single stranded siRNA [69]. Toxicity *in vivo* often results from the immunogenic effects of introducing foreign RNA into cells. Most issues arise as a result of IFN-α production following Toll-like receptor 7/8 activation. These issues can be abrogated by replacing uridines with 2′-O-methyluridine in siRNAs [70]. Additionally, delivery agents may cause harmful side effects because the introduced RNA is trafficked through pathways that expose them to the innate immune system. For example, liposome-mediated delivery of siRNA exposes the RNA to endosomal Toll-like receptors, cytoplasmic RIG-I and protein kinase R (PKR), and other inflammatory pathways [71, 72]. Other delivery agents expose the RNA to a completely different set of pattern recognition receptors, resulting in an interferon response and inflammation [73].

## Therapeutic RNA interference strategies

Many researchers have used RNAi to develop therapeutics against acute and persistent viral infections, and several clinical trials have been carried out. For acute viral infections, siRNA treatments for respiratory syncytial virus (NCT01065935) and influenza (NCT01747148) are currently in clinical trials. These viruses present good targets for RNAi therapeutics because they cause acute infections with pathogenic effects that can be prevented using this approach. In addition, an acute infection is often localized to a single organ or region, requiring only a relatively straightforward delivery approach for RNAi. To date, Alnylam (Cambridge, MA, USA) has successfully conducted phase II clinical trials using the RNAi therapeutic ALN-RSV01, which targets RSV nucleocapsid RNA [74]. Safety and tolerability have been reported, paving the way for future clinical trials to investigate efficacy.

RNAi approaches have also been investigated to treat persistent viral infections such as HIV-1, hepatitis B, and hepatitis C. These are targeted in a different manner to acute infections because the RNA therapy must be present for a longer period of time [75, 76]. The pre-clinical efficacy is promising; nevertheless, a different set of problems are associated with using RNAi for long-term infections and these issues must be further explored before moving to the clinic [77, 78]. The viral proteins and cellular machinery necessary for viral replication are obvious targets for preventing initial infection, but viral targets are susceptible to mutational escape resulting from the relatively low fidelity of the viral RNA polymerase enzymes. Additionally, in the case of HIV-1, cells may be infected with latent virus that can persist for years before viral production, with no or only low levels of viral mRNA being produced during the latent period. Nevertheless, *in vitro* targeting of initial HIV-1 infection has been very successful at inhibiting viral replication and the infection of new cells [79, 80]. Translating the success of RNAi against HIV-1 *in vitro* to an *in vivo* or clinical setting has been hampered by problems with delivery and sustained expression [81, 82]. An optimal delivery agent would efficiently target only those cells with the potential to be infected by HIV-1, or already-infected cells. Current delivery methods do not offer this advantage, so non-specific or *ex vivo* methods are used. Enhancing RNAi expression is also necessary to advance therapeutics to a clinical setting; this can be achieved by increasing the molar ratio of RNAi to delivery reagent or by improving the potency of the RNAi [83]. Alternatively, the HIV-1 promoter can be targeted using transcriptional gene silencing to prevent reactivation of viral production [47, 84]. Peripheral blood mononuclear cells transduced with a lentivirus expressing shRNA against the U3 region of HIV-1 successfully repopulated CD4+ T-cell lineages in mice after HIV-1 infection, suggesting that this approach might be a feasible functional cure [85]. These approaches are very promising, but must be carefully assessed because increasing the concentration of RNAi also increases the possibility of off-target effects and/or the mutation rate [86, 87].

## RNA interference approaches for HIV-1 treatment

RNA-silencing approaches have been designed to control viral replication by blocking key steps within the viral replication cycle [88]. Several considerations must be made when choosing targets for HIV-1 treatment with RNAi (Table 2), including design strategies to target highly conserved regions within viral RNAs or cellular mRNAs that are necessary for viral production [89].

**Table 2:** **Potential RNAi targets for HIV-1 therapy**

	Function of target gene	Type of study	Reference(s)
HIV-1 gene targets			
*Gag*	Proteolytic processing of the HIV-1 genome	Mouse	[95, 168]
*Pol*	Transcription	Mouse	[94]
*env*	Receptor binding and fusion	Phase I/II	[169, 170]
*tat*	Transcription or RNAi modulation	Phase 0	[38]
*rev*	Reverse transcription, integration	Mouse	[140]
*nef*	Immune modulation	Mouse	[92, 171–174]
*pol* (integrase)	Integration	*In vitro*	[91, 175]
*pol* (reverse transcriptase)	Reverse transcription	*In vitro*	[176]
Promoter	Transcription	Mouse	[98]
Long terminal repeats	Genome expression	*In vitro*	[76, 93, 173]
Cellular targets			
CCR5	Receptor binding and fusion	Phase 0/phase I/II	[149]
CXCR4	Receptor binding and fusion	*In vitro*	[120, 177]
CD4	Receptor binding and fusion	*In vitro*	[150, 178]
LEDGF/p75	Integration	*In vitro*	[179]
Importin-7	Integration	*In vitro*	[180]
Chaperonin	Integration	*In vitro*	[181]
P-TEFb	Transcription	*In vitro*	[182]
Tat-SF1	Transcription	*In vitro*	[87]
SPT5	Transcription	*In vitro*	[183]
Cyclin T1	Transcription	*In vitro*	[122]
DDX3	Export	*In vitro*	[123]
SOCS1	Trafficking or immune modulation	*In vitro*	[124]
TRBP	Immune modulation or RNAi pathway	*In vitro*	[75, 125]
TNPO3	Nuclear entry of viral pre-integration complex	Mouse	[140, 151]

### Viral targets

The viral genome (Fig. 1a) consists of several highly conserved regions that have proven to be useful targets as viral escape via mutagenesis is less likely in these regions than in other parts of the genome. To identify highly conserved regions, it is useful to analyze HIV-1 sequences from global clades and primary isolates that are available online from the Los Alamos National Laboratory [90].

Selection of targets for RNAi-based therapies against HIV-1 is a crucial step in obtaining a long-term therapeutic effect without the emergence of resistant strains. Various steps throughout the HIV-1 replication cycle have been targeted, although it can be particularly beneficial to inhibit the early stages of infection (class I targets), such as viral entry, reverse transcription, and integration [2, 91]. Owing to the versatility of RNAi-based therapeutics against any HIV-1 mRNA sequence, it is also possible to target steps of the HIV-1 replication cycle that are not targeted by clinical cART agents (Table 2).

As the unspliced HIV-1 genomic RNA transcripts contain the full sequences for all nine viral genes, it is possible to design anti-HIV-1 siRNAs against any particular conserved coding region. Owing to alternative splicing patterns, however, it can be advantageous to design siRNAs against the common sequences that occur on all spliced and unspliced HIV-1 transcripts, including the early spliced viral mRNAs that contain *tat*, *rev*, and *nef* reading frames. These sequences are among the earliest spliced transcripts of the integrated provirus. Thus, targeting with siRNA can inhibit production of the associated protein and downstream proteins necessary for viral maturation, because the early proteins are required for production of late transcripts. Target sites present in the HIV-1 subgenomic RNAs, including *nef* and the untranslated LTR regions, can be targeted in all spliced and unspliced transcripts. Alterations in the RNA folding and structure of these regions can, however, lead to RNAi-resistance [92, 93]. Alternatively, the targeting of genes that encode the structural and enzymatic proteins required for viral assembly and infection (including *gag*, *pol*, *vif*, and *env*) is effective, despite the fact that these genes are not found in all transcripts [94–97]. While the integrase and reverse transcriptase proteins are targeted by different classes of antiretroviral drugs, the coding regions of these proteins can also be targeted by RNAi. Most approaches have relied on using RNAi to target viral mRNA transcripts in the cytoplasm, but the HIV-1 promoter may also be targeted by transcriptional gene silencing, which induces epigenetic silencing of the integrated provirus [47, 85, 98, 99].

HIV-1 rapidly escapes drug inhibition and RNAi because of its high rate of mutation, so finding highly conserved regions of the HIV-1 genome to target with RNAi is a common strategy. Escape from targeting by mutagenesis at conserved regions may be restricted by a consequent loss of viral fitness. A major goal of developing anti-HIV-1 RNAi therapy is to target HIV-1 in a manner that prevents viral escape; such strategies may include targeting multiple viral sites and endogenous host factors that are required for HIV-1 infection and replication.

Owing to the low fidelity of HIV-1 reverse transcriptase, with an estimated error rate of 3 × 10^−5^ bases per replication [100, 101], the virus can evolve to escape most monotherapies. A variety of mechanisms are responsible for RNAi escape, including single-point mutations (which may disrupt the specific recognition of and binding to the siRNA-binding site), deletion of the target sequence, and transcriptional upregulation [102]. Single-point mutations within the target site of a specific siRNA and mutations that cause structural changes to the mRNA can abate guide-strand recognition of the target mRNA, thereby preventing Ago2-mediated silencing [92, 103–105]. Indirect resistance, such as mutation of non-targeted sites within the viral promoter, can lead to upregulation of viral transcription, thereby allowing viral escape by overwhelming the RNAi-mediated selective pressure [106, 107]. Mutational escape can be avoided by using combination siRNAs that target multiple sites, which may act synergistically to lower the overall required siRNA concentration, or by combining siRNAs with other cART drugs [78, 108]. Like cART, such siRNA-based cocktail approaches increase the threshold for mutational escape, and any HIV-1 escape variants that do occur may also result in a loss of viral fitness [109–111].

### Cellular targets

Targeting the host cellular co-factors that are required for HIV-1 infection is an attractive option for RNAi therapies because endogenous genes are not as prone to mutational escape as viral genes, which must undergo reverse transcription by an error-prone polymerase [75, 112]. The concept of generating an HIV-resistant immune system has been supported by a report on the ‘Berlin Patient’ [113], an individual with HIV-1 infection who required an allogeneic bone marrow transplant for acute myeloid leukemia. The donor hematopoietic progenitor cells (HPCs) were homozygous for a *CCR5* partial gene deletion (*CCR5∆32*). In the absence of the CCR5 co-receptor on the surface of the HPC-derived CD4+ T cells, macrophages, and monocytes from the donor, the CCR5-tropic HIV-1 virus was unable to infect new cells. After the transplant, HIV-1 was no longer present at detectable levels, suggesting that the patient has been functionally cured of HIV/AIDS [114, 115]. This approach is not feasible for the majority of HIV/AIDS patients who do not require bone marrow transplants for pre-existing cancer, but other modes of cellular targeting can be considered. Moreover, a second attempt at an allogeneic transplant of *CCR5∆32* HPCs was not successful because CXCR4-tropic virus became dominant after the interruption of cART [116]. Therefore, it may be necessary to target multiple cellular co-factors, including endogenous genes that are required for cell entry, integration, transcription, and maturation of the virus.

Cellular co-factors that are required for infection include the CD4 receptor as well as the CCR5 and CXCR4 co-receptors. Targeting these receptors can protect the cell from viral entry and infection, but this strategy has limitations depending on the receptor targeted [117, 118]. *CCR5* is an attractive target because it is required for CCR5-tropic viral infection and, because it shares functional redundancy with *CCR2*, it is not required for the normal development and function of T-lymphocytes [119]. By contrast, CXCR4 and CD4 receptors are less favored as targets as they are involved in important cellular processes. CXCR4 is required for T-cell maturation, so disruption of *CXCR4* must be targeted only in mature T cells [118]. Targeting of CD4 is also problematic because of its significant immunological function [120].

Host factors play a role in the integration of the HIV-1 viral genome into cells. Therefore, the HIV-1 replication cycle may be disrupted by using these host factors as targets [75, 121]. Co-factors of integrase, including LEDGF/p75, Importin-7, and Chaperonin, are potential targets [78]. Elongation factors P-TEFb, Tat-SF1, and SPT5 regulate HIV-1 transcription by interaction with the Tat protein [87]. Cyclin T1 interacts with P-TEFb and Tat to initiate HIV-1 transcription [122]. The human RNA helicase DDX3 is responsible for the export of spliced HIV-1 transcripts, and human SOCS1 is responsible for trafficking of HIV-1 *gag* [123]. Additionally, RNAi has been used to inhibit several autophagy inhibitory factors that are responsible for HIV-1 replication and prevention of HIV-1 degradation [121]. As HIV-1 is capable of modulating the immune system through TRBP-TAR-mediated inhibition of *PKR-* or *SOCS1-*dysregulation of immune activation, silencing these genes results in an antiviral response [124]. RNAi silencing of *TRBP* can also inhibit HIV-1 replication [75, 125, 126], but this could potentially disrupt the RNAi pathway because of the role of TRBP in Dicer processing and RISC assembly [127]. In general, cellular targets must be considered with caution because these targets affect cell growth and function, and because they may be important for cell signaling not observed *in vitro*. Another approach is to use a combination of RNAi or other biological agents against cellular and viral targets using gene therapy [128]. There are two clinical trials using this approach [38, 129], which will be discussed in the *ex vivo* delivery section below.

## Delivery

One of the largest hurdles in developing effective gene therapy is delivery. Considerations for the delivery of RNAi include immunogenicity, cell type uptake, and specificity. RNAi-based therapeutics can be delivered as RNA molecules (such as siRNAs or shRNAs), as DNA (plasmids or minicircles), or as therapeutic transgenes in viral vectors [130]. Delivery reagents can be targeted to specific cell types or can be non-specific. The advantage of targeted delivery is that side effects can be reduced, while increasing the efficiency of delivery to target cells. To date, the RNAi-based agents that have reached clinical testing for HIV/AIDS have relied on *ex vivo* delivery of lentiviral vectors into specific cell types, such as CD4+ T cells or CD34+ HPCs, which are then transfused back into the patient [2]. Alternatively, *in vivo* delivery approaches may utilize non-targeted or targeted delivery agents, which include non-viral carriers such as liposomes, dendrimers, and aptamers.

### *Ex vivo* delivery

*Ex vivo* delivery approaches for RNAi-based therapeutics include viral vectors, nanoparticles, and electroporation [131]. The most common of these is *ex vivo* transduction with virus, including adenovirus, adeno-associated virus (AAV), and lentivirus. shRNAs can be encoded in viral vectors to produce stable cell lines and to transduce cells that are refractory to transfection, such as hematopoietic stem cells and T cells [132]. In viral vectors derived from adenovirus, AAV, and lentivirus, shRNA transcripts driven by polymerase III promoters synthesize a single-strand hairpin loop that can be incorporated into the RNAi pathway. Lentivirus is integrative, so the RNAi therapy delivered is incorporated into the cellular genomic DNA, and although the site of insertion is semi-random, lentiviral vectors do not share the same tendency for insertional oncogenesis as gammaretroviral vectors [133]. As lentiviral vectors are constructed using a limited HIV-1 genome, vector production and transduction of the lentiviral vector may be poor when expressing vector-encoded shRNAs that target regions that are common to HIV-1 transcripts and lentiviral vector transcripts, such as the LTR region, or regions that are encoded by helper plasmids, such as *gag*, *pol*, and *rev* [134, 135]. Different strategies can be employed to overcome this restriction by increasing the amount of helper plasmids [135] or by knocking down Ago2 with a separate shRNA [136]. As a ssDNA virus, AAV is mostly non-integrative, although limited integration occurs at a specific locus within cellular genomic DNA. Several non-viral approaches have been taken, including plasmid delivery in liposomes and other nanoparticles, but this results in very inefficient delivery of transcripts to T cells; transcripts that are delivered in this way are also non-integrative, limiting the longevity of their therapeutic value. Other non-specific strategies are non-integrative but also have reduced efficiency compared to lentivirus [137].

The first clinical demonstration of the use of RNAi against HIV-1 was a phase 0 safety and feasibility clinical trial conducted by the City of Hope Hospital (Duarte, CA, USA) and Benitec, Inc. (Sydney, Australia). This study involved transducing human CD34+ HPCs with a self-inactivating and non-replicating lentivirus containing a *tat/rev* shRNA, a TAR decoy, and a *CCR5* ribozyme [38]. Four patients with AIDS-related lymphoma received autologous bone marrow transplants with lentiviral-transduced CD34+ HPCs in an effort to treat both lymphoma and HIV/AIDS. The lentiviral-based therapy was well tolerated and no drug-related adverse events were observed in the four patients. Expression of the shRNA and ribozyme components was detected in the peripheral blood and bone marrow of all four patients for at least 8 months, and for more than 3 years in one patient [38, 39]. More recently, we have developed a next-generation lentiviral construct that expresses three small anti-HIV-1 RNAs from the MCM7 intron and a fourth CCR5 shRNA from the tRNA^Ser^ promoter [136, 138].

Calimmune, Inc. (Los Angeles, CA, USA) has developed a similar strategy using a dual-therapeutic anti-HIV-1 lentiviral vector that expresses a CCR5 shRNA and a C46 peptide, which is derived from the HIV-1 gp41 envelope protein that inhibits viral fusion [139]. In a current phase I/II trial (NCT01734850), the anti-HIV-1 vector is transduced *ex vivo* into autologous CD34+ HPCs and CD4+ T-lymphocytes, which are then transplanted back into the HIV-infected patient [129]. The CCR5 shRNA component is capable of inhibiting CCR5-tropic (R5-tropic) but not CXCR4-tropic (X4-tropic) strains, whereas the C46 peptide inhibits both tropisms of HIV-1 and is thus an essential factor in the dual-therapeutic design of the vector.

### Non-specific *in vivo* delivery

A variety of nanoparticles have been used for encapsulating and delivering HIV-1 siRNAs, including dendrimers, polymers, and liposomes. Using the G5 PAMAM (generation 5 polyamidoamine) dendrimer with three anti-HIV-1 DsiRNAs, HIV-1 replication was potently suppressed in a humanized mouse model [140]. This cocktail strategy combined one DsiRNA against the HIV-1 *tat/rev* overlapping reading frame, another DsiRNA against the host CD4 receptor, and a third DsiRNA against Transportin-3 (TNPO3), which is a host factor required for nuclear entry of the HIV-1 pre-integration complex. Biodegradable polymers can protect siRNAs from ribonuclease degradation but release the siRNAs upon cell entry. This design was used to encapsulate CCR5 siRNAs in a polymer nanocapsule to knockdown levels of CCR5 with significantly greater efficiency than liposome-based delivery systems [141].

Advances in electroporation are creating opportunity for siRNA delivery *in vivo*. RNAi has been delivered to the brain, muscle, and skin [142, 143]. Most recently, Inovio Pharmaceuticals, Inc. (Blue Bell, PA, USA) has developed ELGEN *in vivo* surface electroporation for delivery of siRNA [142] or DNA vaccines to CD4+ memory T cells [144, 145], which is promising because memory T cells are typically thought to be reservoirs for HIV-1. This strategy has yet to be tested with siRNA against HIV-1 targets, and *in vivo* electroporation will need to target a wider range of locations that include other regions of high T-cell concentration, such as the spleen and blood.

### Targeted delivery

Targeted delivery approaches include the use of tissue-specific serotypes of AAV, nucleic acid aptamers, antibodies, and modified nanoparticles. These approaches are non-integrative, but each has its own set of challenges that need to be considered in order to obtain an effective delivery method.

AAV is a DNA virus that is used for gene therapy and that undergoes limited integration into host genomes. AAV serotypes are selected for specificity to certain regions of an organism. For example, AAV2 targets heparan sulfate proteoglycan, α_V_β_5_ integrin and fibroblast growth receptor 1, which is found on hepatocytes, skeletal muscle cells, smooth muscle cells, and neurons [146]. AAV is a very effective vector for gene therapy, but the vector has limited space for exogenous gene insertion and can typically only be delivered once because of its immunogenic properties [147].

Cell-specific nanoparticles comprise another class of targeted delivery molecules that include aptamers, antibodies, and targeted lipid nanoparticles. These particles are very small and often must overcome multiple barriers, such as the cell membrane and endosome, for the siRNA to be loaded into the RISC. Targeted lipid nanoparticles are widely used to deliver siRNA *in vitro*, but can also be applied *in vivo* by conjugating a variety of functionalities to liposomes [71, 148, 149]. These functionalities include proteins, chemical conjugates, and beads. RNAi molecules are encapsulated in a lipid bilayer with targeting molecules facing outwards to bind to receptors of interest.

Aptamers and antibodies selectively deliver siRNAs to cells of interest by binding to surface receptors and then entering the cells by receptor-mediated endocytosis. Unlike some siRNA-nanoparticle designs, aptamers or antibodies do not bestow a protective layer around the siRNAs, so the siRNAs are susceptible to nucleases if not modified. Aptamers are selected through the systematic evolution of ligands by exponential enrichment, and are similar to antibodies in binding affinity and specificity to the target receptor or protein. Several delivery targets for these molecules include the HIV-1 gp120 protein and the CD4 receptor [150–152]. These particles are typically not immunogenic, but are rapidly cleared from the circulation and are non-integrative, so multiple doses of anti-HIV-1 siRNAs must be given [149, 153]. Dimeric bacteriophage pRNA complexes have been engineered with an HIV-1 gp120 aptamer and a *tat/rev* siRNA for targeted delivery to HIV-infected cells [154]. Antibodies have also been used to deliver siRNAs in cell-specific fashion, including *gag* siRNAs complexed with a protamine-gp160 antibody fusion protein [155] and siRNAs against the CCR5 co-receptor and viral genes *tat* and *vif* conjugated with poly-(D)-arginine and a CD7 antibody [156].

Microbicides loaded with siRNAs or aptamer-siRNAs are designed to prevent the transmission of the virus, and their efficacy is increased by targeting localized areas. CD4 aptamer-siRNA chimeras can be administered topically as microbicides for prophylaxis against HIV-1 [150, 157]. In other systems, the siRNA is either naked or conjugated to cholesterol and applied topically to the skin, and results in prevention of viral transmission [158]. For complete and effective HIV-1 prophylaxis, however, siRNA microbicides must achieve durable protection from HIV-1, broad target specificity against potential viral variants, and favorable kinetics for cellular uptake and virus neutralization.

## Conclusions

The potential for RNAi to be used against HIV-1 in the clinic is feasible with improvements in current strategies. Several points to consider when using RNAi to treat HIV-1 include delivery, immunogenicity, toxicity, and viral mutagenesis. In this review, we have discussed strategies that are used to overcome problems in each of these areas. Modified base incorporation and improved siRNA specificity have led the way in reducing the immunogenicity and improving efficacy. In addition, target identification has produced a wide array of viral and cellular targets that inhibit HIV-1 replication upon silencing. As a therapeutic, siRNAs have great promise due to their target specificity, potency, and ability to be chemically synthesized for manufacturing. They are widely adaptable, which is advantageous in the case of HIV-1 mutagenesis. By developing a platform for clinical use of RNAi against HIV, targets can be changed rapidly as the virus mutates.

The mode of delivery remains one of the highest hurdles in bringing RNAi to the clinic to treat HIV. To have an effective siRNA therapeutic, delivery must efficiently reach the intended cells and tissues. To date, the most efficient delivery methods are viral vectors, including the *ex vivo* transduction of cells with lentivirus and *in vivo* delivery with AAV. Unfortunately, these delivery types are only moderately specific, and AAV is prone to inducing an immunogenic response *in vivo*. More targeted delivery methods are applicable in an *in vivo* setting, where they offer specificity and reduced immunogenicity; nevertheless, these methods often lack the pharmacokinetics and scalability of non-specific delivery carriers. Therefore, continued progress to improve each of these shortcomings is required to fulfill the promise of RNAi therapeutics for HIV.

## Abbreviations

AAV: adeno-associated virus
Ago2: Argonaute-2
AIDS: acquired immunodeficiency syndrome
cART: combinatorial antiretroviral therapy
DsiRNA: Dicer substrate RNA
gp120: glycoprotein 120
HIV-1: human immunodeficiency virus-1
HPC: human progenitor cells
IFN: interferon
LTR: long terminal repeat
miRNA: microRNA
PKR: protein kinase R
RISC: RNA-induced silencing complex
RNAi: RNA interference
shRNA: short hairpin RNA
siRNA: short interfering RNA
vsiRNA: viral suppressors of RNAi
VSR: viral suppressors of RNAi
vsRNA: viral small RNA
ZFN: zinc finger nucleases
